# Two novel heterozygous truncating variants in *NR4A2* identified in patients with neurodevelopmental disorder and brief literature review

**DOI:** 10.3389/fnins.2022.956429

**Published:** 2022-08-03

**Authors:** Xiaozhen Song, Wuhen Xu, Man Xiao, Yanfen Lu, Xiaoping Lan, Xiaojun Tang, Nanjie Xu, Guangjun Yu, Hong Zhang, Shengnan Wu

**Affiliations:** ^1^Molecular Diagnostic Laboratory, Department of Clinical Laboratory, Shanghai Children’s Hospital, School of Medicine, Shanghai Jiao Tong University, Shanghai, China; ^2^Department of Neurology, Shanghai Children’s Hospital, School of Medicine, Shanghai Jiao Tong University, Shanghai, China; ^3^Research Center of Translational Medicine, Shanghai Children’s Hospital, School of Medicine, Shanghai Jiao Tong University, Shanghai, China; ^4^Department of Anatomy and Physiology, School of Medicine, Shanghai Jiao Tong University, Shanghai, China; ^5^Shanghai Children’s Hospital, School of Medicine, Shanghai Jiao Tong University, Shanghai, China

**Keywords:** *NR4A2*, truncating, neurodevelopmental disorder, intellectual disability, language impairment, attention deficit

## Abstract

Pathogenic variants in the nuclear receptor superfamily 4 group A member 2 (*NR4A2*) cause an autosomal dominant neurodevelopmental disorder with or without seizures. Here, we described two patients presenting with developmental delay, language impairment, and attention-deficit hyperactivity disorder. Trio-based whole exome sequencing revealed two novel heterozygous variants, c.1541-2A > C and c.915C > A, in *NR4A2*. Both variants were identified as *de novo* and confirmed by Sanger sequencing. *In vitro* functional analyses were performed to assess their effects on expression of mRNA or protein. The canonical splicing variant c.1541-2A > C caused aberrant splicing, leading to the retention of intron 7 and a truncated protein due to an early termination codon within intron 7 with decreased protein expression, while the variant c.915C > A was shown to result in a shorter protein with increased expression level unexpectedly. The clinical and genetic characteristics of the previously published patients were briefly reviewed for highlighting the potential link between mutations and phenotypes. Our research further confirms that *NR4A2* is a disease-causing gene of neurodevelopmental disorders and suggests alterations in different domains of *NR4A2* cause various severity of symptoms.

## Introduction

The extensive clinical use of exome sequencing has enabled increasing recognition of the genetics and pathophysiology of neurodevelopmental disorders (NDD), in which *de novo* variants play major roles (Retterer et al., 2016). The nuclear receptor superfamily 4 group A member 2 (*NR4A2*) gene, also known as nuclear receptor-related 1 (*Nurr1*), is located on chromosome 2q24.1 and encodes an orphan nuclear receptor that belongs to the nuclear steroid-thyroid hormone and retinoid receptor superfamily (Zetterstrom et al., 1997; Wang et al., 2003). *NR4A2* is widely expressed throughout the brain and in other tissues and organs, such as the cortex, hippocampus, and peripheral blood. The encoded protein is a transcription factor with essential regulatory functions in the central nervous system (CNS), including differentiation, migration, maturation, and maintenance of mesencephalic dopaminergic neurons, which are related to memory and learning. It has been shown that *NR4A2* knockout mice are unable to produce midbrain dopaminergic neurons and die shortly after birth (Safe et al., 2016).

In human pathology, earlier studies have indicated *NR4A2* may be a potential susceptibility gene for Parkinson’s disease and schizophrenia by case-control association studies. The G insertion at IVS6 + 18 of *NR4A2* was observed with higher frequency in Parkinson’s patients than in healthy individuals (Zheng et al., 2003; Grimes et al., 2006; Chen et al., 2007; Liu et al., 2013). Le et al. (2003) reported two mutations −291Tdel and −245T > G located in the non-coding exon 1 of the *NR4A2* gene in 10 of 107 individuals with familial Parkinson’s disease, but not in unaffected controls. Besides mutations in non-coding or intronic regions, one rare missense mutation (c.709C > G) in exon 3 of *NR4A2* was detected in a patient with Parkinson’s disease (Grimes et al., 2006). Additionally, missense mutations (c.289A > G and c.308A > G) in exon 3 of *NR4A2* were identified in schizophrenic patients, and a small deletion mutation (c.364_366delTAC) in an individual with manic-depressive disorder (Buervenich et al., 2000). Subsequently, *de novo* heterozygous deletions in 2q24.1 with the minimum of overlap being *NR4A2*, yielded by the application of array comparative genomic hybridization (array-CGH) and Next-Generation Sequencing (NGS), have been described in patients with intellectual disability (ID), language impairment and autism spectrum disorder (ASD), suggesting the role of *NR4A2* haploinsufficiency in NDD (Barge-Schaapveld et al., 2013; Leppa et al., 2016; Reuter et al., 2017; Shimojima et al., 2017; Levy et al., 2018). However, *NR4A2* has been considered as a monogenic disease-causing gene due to the recent genetic findings. The *NR4A2* loss-of-function variant c.326dupA was identified in a patient with epilepsy, ID, and language impairment (Ramos et al., 2019). The clinical features of mild intellectual disability at childhood and dystonia parkinsonism in early adulthood observed in two patients have been attributed to the frameshift variants in *NR4A2* (c.326dupA and c.881dupA) (Wirth et al., 2020). Eight missense and loss-of-function variants within *NR4A2* identified in a larger cohort consolidated the pivotal role of *NR4A2* in NDD (Singh et al., 2020). To date, all reported patients carrying *NR4A2* likely pathogenic/pathogenic (LP/P) variants presented with certain consistent neurodevelopmental features, including varying levels (mild to severe) of ID/developmental delay (DD), language impairment, behavioral problems, movement disorders, and epilepsy.

Here, we described two novel *NR4A2* truncating variants in two unrelated Chinese patients mainly presenting with mild ID/DD, language impairment, and attention deficit, without epilepsy. The results of our study provided more evidence for the role of *NR4A2* haploinsufficiency in NDD and updated the mutation spectrum of *NR4A2*. Meanwhile, we briefly reviewed the *NR4A2* variants and clinical manifestations and found that loss-of-function variants and missense variants occurring in the key domains of NR4A2 were generally intolerant.

## Materials and methods

### Patients

Two patients from unrelated families enrolled at Shanghai Children’s Hospital were included in this study. Peripheral blood samples and clinical data from each patient and the parents were collected. Written informed consent was obtained from the parents for the use of clinical and genetic information. This study was approved by the Ethics Committee of Shanghai Children’s Hospital (2020R007-F01).

### Genetic analysis

To elucidate the cause of the disorder, we performed child–parent trio whole-exome sequencing (TWES) on the two patients and their parents due to the known efficiency of TWES for NDD. Genomic DNA for TWES and subsequent Sanger sequencing was extracted from peripheral blood using a DNA Blood Mini Kit (QIAGEN, Germany), following the manufacturer’s protocol. An IDTxGen^®^ Exome Research Panel (IDT, United States) was used to capture the exons, and HiseqX10 (Illumina, United States) was used to sequence the DNA fragments. FASTQ data analysis was conducted as described in previous studies (Xiaozhen et al., 2021). The candidate pathogenic genes were confirmed by Sanger sequencing. The pathogenicity of the variations was evaluated according to the American College of Medical Genetics and Genomics and Association for Molecular Pathology (ACMG/AMP) guidelines and ClinGen specifications (Richards et al., 2015; Zhang et al., 2020).

### Plasmids construction and functional studies of the variants in NR4A2

Wild type (WT) and mutant human *NR4A2* (transcript NM_006186.3) expression plasmids, labeled with an EGFP tag at the N-terminal, were synthesized and cloned into the pCMV-EGFP-C1 vector. The *NR4A2* mutation c.915C > A (p.Cys305*) was introduced into the WT isoform (named NR4A2-WT) using a site-directed mutagenesis kit (Santa Clara, CA, United States). To determine the effects of variant c.1541-2A > C on splicing, WT (c.1541-2A) or mutant (c.1541-2C) intron 7 containing 149 nucleobases was introduced into NA4A2-WT isoform between exon 7 and exon 8. The successfully constructed recombinant plasmid products were named NR4A2-AG and NR4A2-CG (Supplementary Figure 1), respectively. The primers used in the study are shown in Supplementary Table 1. Human embryonic kidney (HEK)-293T cells were transfected with different expression plasmids. The pCMV-EGFP-C1 vector expressing fluorescence was used as a transfection marker.

Forty-eight hours after transfection, total RNA was isolated from cultured cells using TRIzol Reagent (Thermo Fisher Scientific, Waltham, MA, United States), and 1 μg of RNA was converted to cDNA by reverse transcription using EasyScript One-Step gDNA Removal and cDNA Synthesis SuperMix (TransGen Biotech, Beijing, China). The PCR amplification of cDNA, including the target region spanning exons 6–8, was performed using DNA Polymerase (TransGen Biotech, Beijing, China). The primers were as follows: F-TGGAGATGACACCCAGCATA; R-GTGGCACCAAGTCTTCCAAT. Agarose gel electrophoresis (1%) and PCR products’ sequencing were performed to evaluate the products’ length and sequence.

Total protein was extracted using a Protein Extraction Kit (Sangon Biotech, Shanghai, China). Subsequently, 25 μg of total protein was electrophoresed on 8% sodium dodecyl sulfate polyacrylamide gels (SDS-PAGE) and then transferred to nitrocellulose membranes (Merck Millipore, Darmstadt, Germany). After blocking with 5% skim milk, the membranes were incubated with specific primary antibodies overnight at 4°C, followed by incubation with HRP-conjugated anti-mouse (ZSGB-BIO, Beijing, China) secondary antibodies at room temperature for 1 h. Finally, the bands were detected using ECL Prime Western Blotting Reagent (GE Healthcare, Buckinghamshire, United Kingdom). The experiments were repeated in triplicate after determining the optimal working conditions. The primary antibodies used were rabbit anti-GAPDH (Cell signaling technology; 1:3,000) and mouse anti-Nurr1binding to amino acids 2–99 of human Nurr1 (Abcam; 1:500).

### Cell culture and plasmid transfection

HEK-293T cells (purchased from the National Infrastructure of Cell Line Resource, China) were plated at a density of 5 × 10^5^ cells into six-well culture plates. The cells were maintained in Dulbecco’s modified eagle’s medium (DMEM; Gibco, United States), supplemented with 1% penicillin/streptomycin (Gibco, United States) and 10% fetal bovine serum (FBS; Gibco, United States), at 37°C in a humidified atmosphere incubator with 5% CO_2_, and passaged every 2–3 days. Transient transfection with WT or variant *NR4A2* plasmids was performed using Lipofectamine 2000 Transfection Reagent (Thermo Fischer Scientific, United States) according to the manufacturer’s recommendations. Briefly, the day before transfection, cells were plated at a density of 5 × 10^5^ cells per well; 24 h later, 2 μg/well plasmid DNA was added, diluted with 100 μl Opti-MEM (Gibco, United States), and mixed with 6 μL of Lipofectamine 2000.

## Results

### Clinical description

The patients recruited in this study were the only children of their families and were born at full term to non-consanguineous parents with uneventful pregnancies and deliveries. Physical examinations found no dysmorphic features. Neither of them had a remarkable family history of neurologic diseases.

Patient 1 (P1) was an 11-year-old boy at the time of his visit to the clinic. His head circumference and length at birth were within the normal range, and his birth weight was in the 90th percentile. Motor development was in normal milestones with walking independently at 14 months. Significant delay of speech development was observed at the age of 6 years. He presented with a lack of expressive language and poor oral communication with other children. He showed behavioral abnormalities, including attention deficit hyperactivity disorder (ADHD), learning difficulties, tantrums, and aggression. He had mild hypotonia. Both electroencephalogram (EEG) activity and brain MRI revealed no abnormalities. He had no history of seizures.

Patient 2 (P2) was a 12-year-old boy. His developmental milestones were globally delayed, and his fine motor development was poor. He displayed ADHD, learning difficulties, and mild hypotonia. His brain MRI was normal. He had normal EEG and no history of seizures.

### Genetic results

**FIGURE 1:**
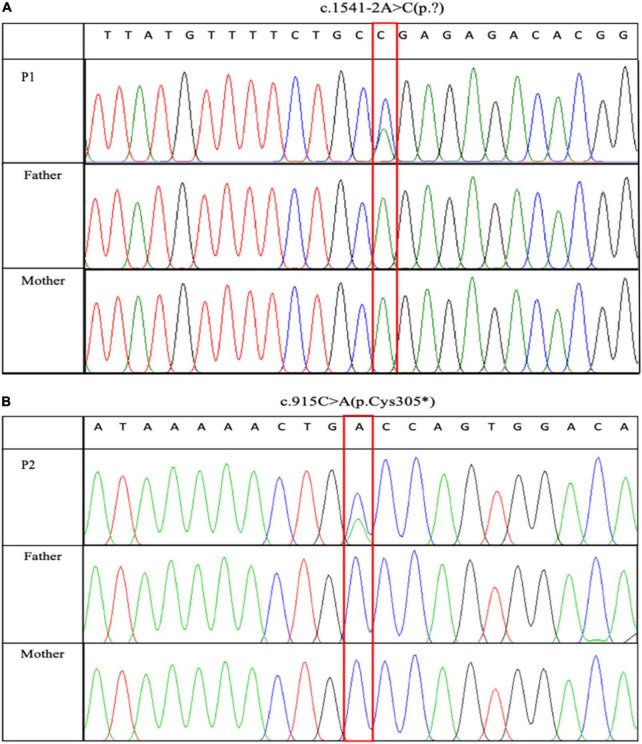
Sanger sequencing maps of two pathogenic variants. c.1541-2A > C and c.915C > A were identified in P1 and P2, respectively. Sanger sequencing verified that the two variants occurred *de novo*. **(A)** c.1541-2A > C was identified in P1. **(B)** c.915C > A was identified in P2.

Child-parent trio-based whole-exome sequencing revealed two novel *NR4A2* variants, including a heterozygous canonical splice-acceptor variant in intron 7, NM_006186.3: c.1541-2A > C in patient 1 (Figure 1A), and a heterozygous nonsense variant within exon 4, NM_006186.3: c.915C > A (p.Cys305*) in patient 2 (Figure 1B), respectively; both variants occurred *de novo* (Figure 1). Neither of the variants was present in the Genome Aggregation Database (gnomAD). The variant c.1541-2A > C detected in P1 was predicted by *in silico* tools to affect splicing and potentially lead to a deficiency in NR4A2 protein. The nonsense variant, c.915C > A, introducing a premature termination, was predicted to lead to either a truncated non-functional protein or a nonsense-mediated mRNA decay (NMD). No other potentially LP/P variants were found.

### Functional assay for the c.1541-2A > C and c.915C > A variants in NR4A2

**FIGURE 2:**
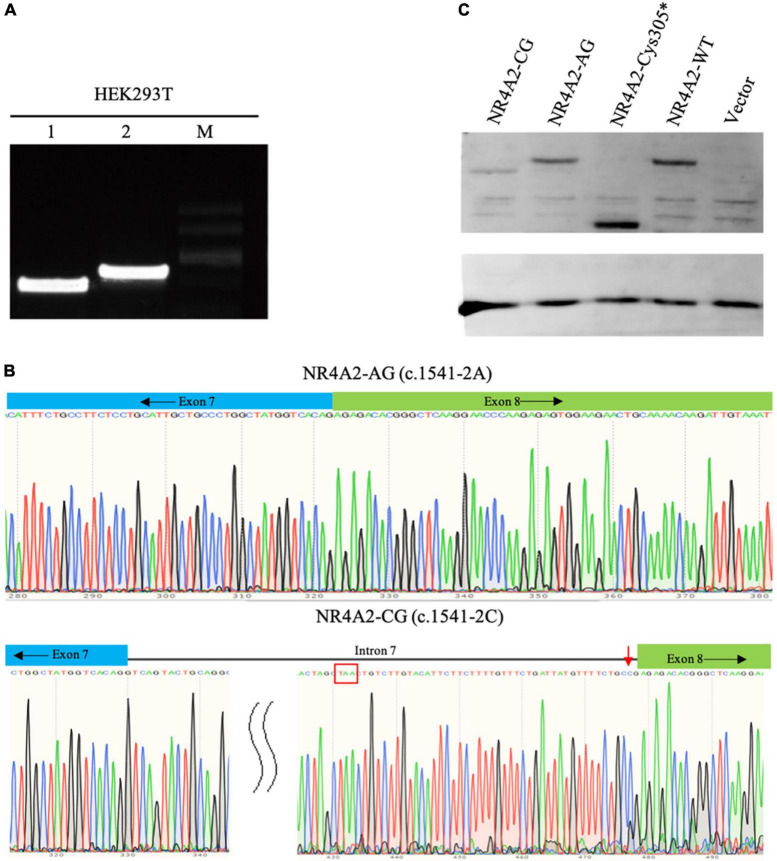
Functional analyses of two *NR4A2* variants: c.1541-2A > C and c.915C > A. **(A)** Agarose gel electrophoresis analysis of the RT-PCR amplification products: 1-NR4A2-AG construct; 2-NR4A2-CG construct; M-marker. **(B)** Sequence analyses of the PCR amplification products of the target fragment of NR4A2-AG and NR4A2-CG. NR4A2-CG resulted in the retention of intron 7 and an early termination codon TAA (red box). **(C)** Protein expression analyses of NR4A2 with c.915C > A or c.1541-2A > C variants. Both c.915C > A and c.1541-2A > C caused a truncated NR4A2 protein.

The *NR4A2* gene contains eight exons in total, and the c.1541-2A > C variant is located at the splicing acceptor site of intron 7. Due to the unavailability of fresh blood or cell samples from patient 1 with c.1541-2A > C variant, we constructed an *NR4A2* recombinant plasmid, introducing intron 7 of *NA4A2* between exon 7 and exon 8 in *NR4A2* WT isoform. The recombinant plasmids consisting of exons 1–7 and intron 7, followed by exon 8, were successfully constructed and were confirmed by plasmid sequencing (Supplementary Figure 1). The functional analyses demonstrated that the mutant NR4A2-CG resulted in a longer PCR product than the wild-type NR4A2-AG, which was confirmed by sequencing (Figure 2A). We found that the NR4A2-CG caused aberrant splicing and produced a longer transcript with the retention of intron 7 (Figure 2B). Additionally, an early termination codon was detected in intron 7 (Figure 2B), which may finally produce a premature truncated protein.

To verify the effect of two detected variants c.1541-2A > C and c.915C > A (p.Cys305*) on protein expression, western blot studies were performed. The protein analyses revealed different molecular weight patterns in the cells transfected with different plasmids. The c.1541-2A product (NR4A2-AG) and NR4A2-WT produced the protein with the same size, while both variants c.915C > A and c.1541-2A > C ended in smaller molecular weight pattern compared to that of the wild type (Figure 2C). This indicated that the two variants produced truncated NR4A2 proteins, resulting in the loss of key protein domains, which could potentially cause the loss of function (LoF) of NR4A2. The variant c.1541-2A > C caused decreased protein expression compared to the wild type, as expected. Unexpectedly, we found that the nonsense variant c.915C > A (p.Cys305*) led to higher protein expression than the wild type, which could be attributed to the increased stability and lower degradation of the truncated protein.

### Brief literature review about NR4A2 variants and clinical manifestations

**FIGURE 3:**
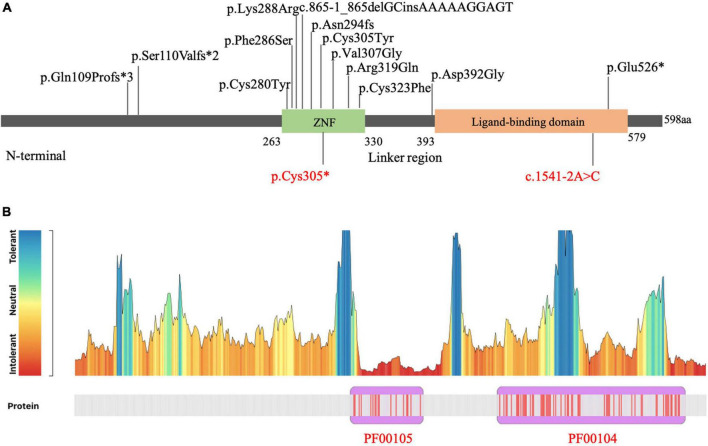
Distribution of *NR4A2* variants. **(A)** Schematic view of the domain distribution of variants in *NR4A2.* The variants identified in the present study (in red) and previous studies (in black). **(B)** *NR4A2* missense variation tolerance landscape. The tolerance landscape depicts a missense over synonymous ratio which are annotated from gnomAD dataset. Pkinase Pfam protein domains are shown in purple. PF00105 is clearly seen as intolerant to missense variation compared to other parts of NR4A2.

To date, sixteen patients carrying *NR4A2* LP/P single nucleotide variants (SNVs), or small insertion-deletion (InDel) have been reported (Vissers et al., 2017; Ramos et al., 2019; Singh et al., 2020; Wirth et al., 2020; Jesus et al., 2021; Winter et al., 2021). The main clinical features and genetic information of patients with *NR4A2* LP/P variants are summarized in Table 1. The main manifestations are characterized by ID, language impairment, seizures, psychobehavioral problems, and movement disorders. No significant difference was observed in age of onset and phenotypic severity between patients with LP/P missense variants and loss-of-function variants. The majority of the patients with *NR4A2* variants presented with ID and language impairment ranging from mild to severe. A schematic view of the distribution of LP/P variants in NR4A2 protein domain is shown in Figure 3A, including eight missense variants and seven loss-of-function variants. NR4A2 protein consists of an N-terminal, a DNA binding domain (DBD), a ligand binding domain (LBD), and a linker region between DBD and LBD. DBD domain contains two zinc fingers. All the pathogenic missense variants were found to occur in the zinc finger domain with the exception of p.Asp392Gly, which was located in the linker region adjacent to LBD. Loss-of-function variants have been observed to occur in any region of NR4A2 protein. We performed MetaDome analysis which using data from gnomAD and ClinVar for evaluating the tolerance of *NR4A2* missense variants^1^ (Wiel et al., 2019). The *NR4A2* tolerance landscape has shown that NR4A2 protein contains two key homologous domains, Pkinase Pfam protein domain PF00105 and PF00104. The PF00105 (consistent with zinc finger domain in Figure 3A) is intolerant compared to other regions in NR4A2 protein, which helps to understand why missense variants occurring in this region are more likely damaging (Figure 3B).

**TABLE 1:** Genetic and clinical features of patients with *NR4A2* LP/P variants.

Patient	References	Variant	Variant type	Sex/Age (Y)	Seizures	Intellectual disability	Language impairment	Psychobehavioral problems	Movement disorders
1	Vissers et al. (2017)	c.920T > G, p.Val307Gly	Missense	F/15	No	Psychomotor retardation	NA	NA	No
2	Ramos et al. (2019)	c.326dupA, p.Ser110Valfs*2	Frameshift	NA	Yes	Mild	Yes	NA	No
3	Wirth et al. (2020)	c.326dupA, p.Ser110Valfs*2	Frameshift	M/29	Yes	Mild	Yes	No	Adult-onset dystonia- parkinsonism
4	Wirth et al. (2020)	c.881dupA, p.Asn294fs	Frameshift	F/57	No	Mild	Yes	No	Adult-onset dystonia- parkinsonism
5	Singh et al. (2020)	c.839G > A, p.Cys280Tyr	Missense	F/15	Yes	Severe	NA	Autism	No
6	Singh et al. (2020)	c.865-1_865delGCinsAAA AAGGAGT, p.?	Splicing	M/12	Yes	Mild	Yes	Hyperactivity, anxiety	Joint hypermobility in the setting of hypotonia
7	Singh et al. (2020)	c.914G > A, p.Cys305Tyr	Missense	F/9	Yes	Mild to moderate	NA	NA	Dystonia, choreathetoid movements, ataxic gait
8	Singh et al. (2020)	c.1175A > G, p.Asp392Gly	Missense	F/3	Yes	Severe	NA	No	Dystonia
9	Singh et al. (2020)	c.1576G > T, p.Glu526*	Non-sense	M/5	No	Mild	Yes	Attachment disorder, hyposensitivity	No
10	Singh et al. (2020)	c.325dupC, p.Gln109Profs*3	Frameshift	M/2	Yes	NA	Yes	Sensory sensitivity	No
11	Singh et al. (2020)	c.857T > C, p.Phe286Ser	Missense	F/4	No	Moderate	Yes	No	No
12	Singh et al. (2020)	c.968G > T, p.Cys323Phe	Missense	F/19	No	Moderate to severe	Yes	No	No
13	Jesus et al. (2021)	c.956G > A, p.Arg319Gln	Missense	M/30	No	Mild	Yes	Attention deficit	Dystonia-parkinsonism
14	Winter et al. (2021)	c.863A > G, p.Lys288Arg	Missense	M/2.5	NA	Mild to moderate	Yes	No	Early-onset dystonia
15	Present study	c.915C > A, p.Cys305*	Non-sense	M/12	No	Mild	Yes	Attention deficit, hyperactivity, aggression	No
16	Present study	c.1541-2A > C, p.?	Splicing	M/11	No	Mild	Yes	Attention deficit	No
Total					7/16	15/16	12/16	7/16	7/16

F, female; M, male; Y, year; NA, not available.

## Discussion

*NR4A2* is ubiquitously expressed in subcellular regions of the human brain and is particularly prominent in the hippocampus, on which memory-inducing activities, such as language development and learning, are dependent (Abrahams et al., 2007; Hawk and Abel, 2011). Microdeletions encompassing *NR4A2* and point mutations resulting in haploinsufficiency in *NR4A2* have been previously reported in individuals with ID and language impairments, suggesting the role of *NR4A2* in human NDD (Reuter et al., 2017; Levy et al., 2018; Ramos et al., 2019). A recent genetic study with larger cohort identified eight *de novo* variants of *NR4A2* and a deletion containing *NR4A2* in nine patients with NDD and epilepsy, further strengthening the association of *NR4A2* heterozygous variants with NDD (Singh et al., 2020). Here, we reported two novel variants occurring *de novo* in *NR4A2*, c.1541-2A > C and c.915C > A (p.Cys305*), in two patients presenting with mild intellectual disability, language impairment, and attention deficit disorder. Our findings in the patients of Chinese origin provided additional evidence supporting *NR4A2* as a disease-causing gene of NDD. The first patient also presented with behavioral problems, including tantrums, aggression, and hyperactivity, which is consistent with the phenotypes related to *NR4A2* haploinsufficiency described previously (Singh et al., 2020). Epilepsy, a commonly phenotype described in *NR4A2*-associated patients, was not observed in our patients. In previous studies, variants in *NR4A2* have also been associated with early-onset dystonia parkinsonism (Wirth et al., 2020; Jesus et al., 2021; Winter et al., 2021). Although our pediatric patients have not yet presented with movement disorders, these phenotypes should be monitored carefully in future clinical follow-ups.

The variant c.1541-2A > C in *NR4A2* was predicted to lead to aberrant splicing. The mechanism by which it affected *NR4A2* pre-mRNA splicing was confirmed by *in vitro* analysis. The PCR and Sanger sequencing results showed that the c.1541-2A > C variant abolished the splice acceptor site, retaining intron 7 and leading to a premature termination at 104 bp of intron 7, resulting in a truncated protein with 547 amino acid residues compared to the normal protein with 598 amino acid residues. This variant was predicted to affect the expression of the NR4A2 protein, which was partially confirmed by our experiments.

Our experimental studies using HEK-293T cells demonstrated that the variant c.1541-2A > C resulted in a smaller NR4A2 protein with decreased expression which NMD might underlie. The variant c.915C > A produced a significantly truncated protein with increased expression level unexpectedly, suggesting a dominant-negative effect on wild-type protein beyond haploinsufficiency. However, the NR4A2 protein, as an orphan nuclear receptor, has two main domains: a DBD and an LBD (de Vera et al., 2019). The variant c.915C > A was located in the DBD and resulted in the loss of DBD and the downstream domain LBD, indicating that the truncating variant potentially causes loss of the ability of NR4A2 protein binding DNA elements and subsequent functions.

Thus far, a total of 636 variants of *NR4A2* have been recorded in the gnomAD database,^2^ including nine heterozygous variants with loss of function mutations (two stop-gaining, four frameshift, and three splicing), presenting with extremely low frequencies in the assumed healthy population, ranging from 3.98 × 10^–6^ to 3.07 × 10^–5^ with no presence of homozygotes. The pLI (probability of loss of function intolerance) and LOEUF (loss-of-function observed/expected upper bound fraction) scores reported for *NR4A2* were 1.0 and 0, respectively, suggestive of the intolerance of haploinsufficiency due to truncating variants and whole gene deletion, which have been shown to cause NDD. The majority of truncating mutations potentially cause loss of the encoded protein mainly by NMD. *NR4A2* deficiency was previously associated with impaired dopaminergic function and increased the vulnerability of midbrain dopaminergic neurons (Jankovic et al., 2005). The heterozygous deletion of *NR4A2* in mice led to a progressive loss of dopaminergic neurons in the substantia nigra (Jiang et al., 2005) and the occurrence of parkinsonian features (Zhang et al., 2012). The truncated NR4A2 protein perhaps causes NDD by damaging midbrain dopaminergic neurons.

Meanwhile, *NR4A2* also has a substantial Z score for missense variants (Z = 2.24), indicating a certain degree of intolerance to missense mutations. *NR4A2* missense variants considered to cause NDD occurred in DBD and LBD regions, while others occurring in N-terminal domain (c.289A > G, c.308A > G and c.374C > G) tend to be associated with susceptibility of Parkinson’s disease or schizophrenia, in which a different mechanism might play a role. NR4A2 is known to function as a homodimer (Philips et al., 1997). Missense variants located at the functional domains might cause loss of NR4A2 via dominant negative effect. In addition, gain of function resulted from missense variants of *NR4A2* could not be excluded. Further studies to clarify various disease-causing mechanism contributing to different clinical phenotypes are needed.

## Conclusion

In summary, we confirmed that both splicing site variant c.1541-2A > C and nonsense variant c.915C > A detected in our patients caused the truncation of the protein based on functional assays *in vitro*. To clarify the correlations between *NR4A2* variants and clinical presentations, which, thus far, are mainly relevant for NDD, additional patients of different ages and ethnic backgrounds need to be described. Our findings confirmed that *NR4A2* haploinsufficiency was responsible for certain clinical features in patients and proposed the potential appearance of dystonia and/or parkinsonism related to *NR4A2* which should be attached importance to in the follow-ups.

## Data availability statement

The datasets presented in this study can be found in online repositories. The names of the repository/repositories and accession number(s) can be found in the article/Supplementary material.

## Ethics statement

The studies involving human participants were reviewed and approved by the Ethics Committee of Shanghai Children’s Hospital. Written informed consent to participate in this study was provided by the participants’ legal guardian/next of kin. Written informed consent was obtained from the minor(s)’ legal guardian/next of kin for the publication of any potentially identifiable images or data included in this article.

## Author contributions

XS and SW designed the study. XS and MX performed *in vitro* studies. WX and YL collected and evaluated clinical presentations. XL and XT performed NGS and Sanger sequencing. NX and GY provided critical feedback and helped conduct research. XS and WX drafted the manuscript. HZ and SW supervised the study and revised the manuscript. All authors discussed the final results, critically reviewed the manuscript, read, and agreed to the published version of the manuscript.

## Footnotes

1
https://stuart.radboudumc.nl/metadome

2
https://gnomad.broadinstitute.org/
