# Supplementary material for: Two novel heterozygous truncating variants in NR4A2 identified in patients with neurodevelopmental disorder and brief literature review
Source: Front Neurosci. 2022 Aug 3;16:956429. doi: 10.3389/fnins.2022.956429 (PMC9383035; doi:10.3389/fnins.2022.956429)
Supplement: Supplementary file 1 [file Data_Sheet_1.DOCX]

Supplementary Material

Table S1. Primers used in this study

| RT-PCR-F | TGGAGATGACACCCAGCATA |
| --- | --- |
| RT-PCR-R | GTGGCACCAAGTCTTCCAAT |
| Sanger sequencing-c.915C>A-F | ACAGCAAGGAGCGGCAAGGTT |
| Sanger sequencing-c.915C>A-R | GTGGAACGTGATGCTGGAGTATG |
| Sanger sequencing-c.1541-2A>C-F | GCAATGCGTTCGTGGCTTTGG |
| Sanger sequencing-c.1541-2A>C-R | GAGACTGCTCACACGGCTATC |
| pEGFP-NR4A2-WTPlasmid-F | agctgtacaagtccggactcagatctcgagacatgccttgtgttcaggcgcagtat |
| pEGFP-NR4A2-WTPlasmid-R | atgatcagttatctagatccggtggatccttagaaaggtaaagtgtccaggaaaagtttg |
| pEGFP-NR4A2-c.915APlasmid-F | agcaaataaaaactgaccagtggacaagcgtc |
| pEGFP-NR4A2-c.915APlasmid-R | acgcttgtccactggtcagtttttatttgcta |
| pEGFP-NR4A2-AG-F | gattatgttttctgcagAGAGACACGGGCTCA |
| pEGFP-NR4A2-AG-R | GAGCCCGTGTCTCTctgcagaaaacataatca |
| pEGFP-NR4A2-CG-F | GAATGGATTGATTCCATTGTTGAATTCTCCTCCAACTTGCAG |
| pEGFP-NR4A2-CG-R | gatcagttatctagatccggtggatccTTAGAAAGGTAAAGTGTCCAGG |


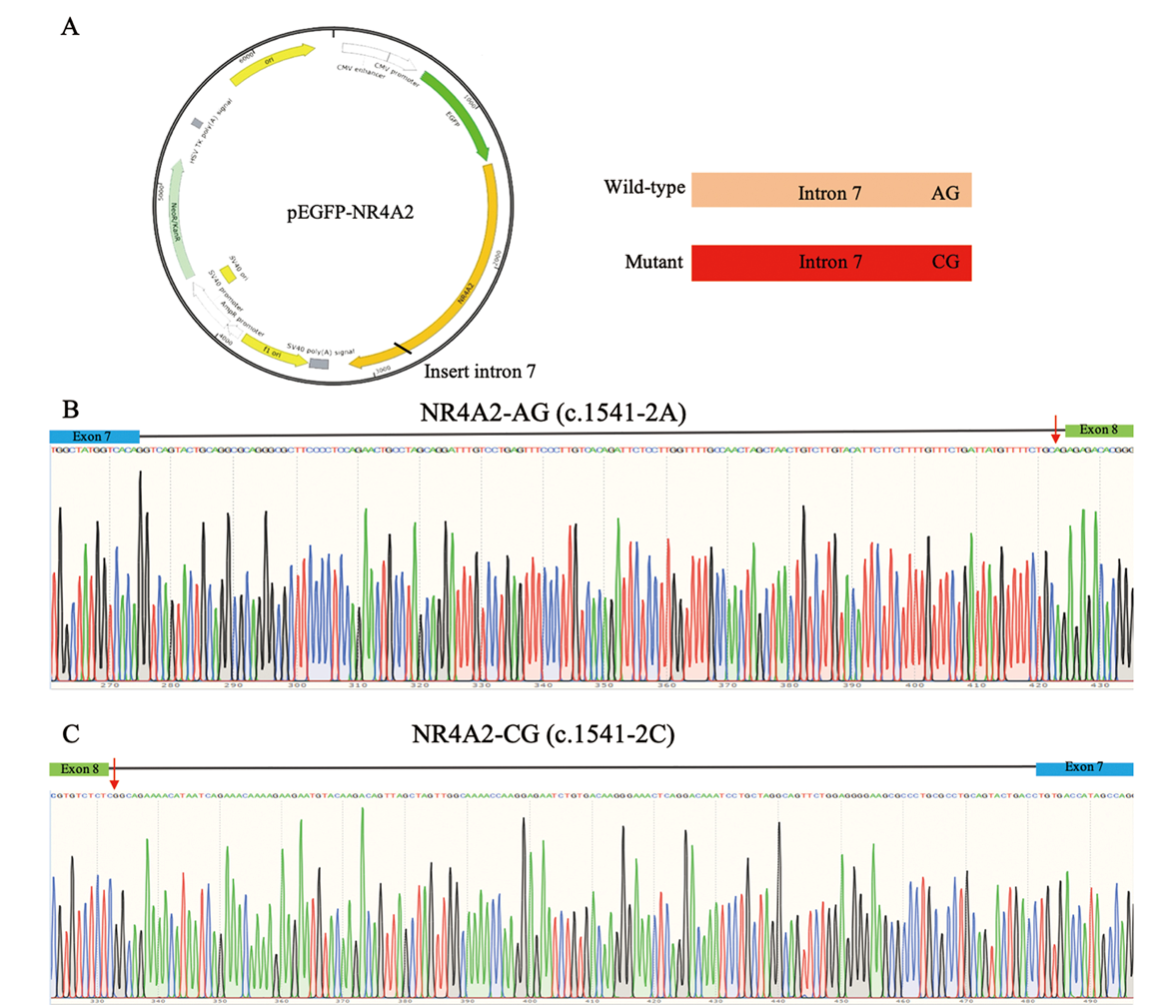


**Figure S1.** Structure of recombinant plasmid used to examine the effect of splicing variant c.1541-2A>C. (A) The vector pCMV-EGFP-C1 and *NR4A2* containing WT or mutant intron 7. (B) Sequencing results of the target fragment of the NR4A2-AG in the positive direction. (C) Sequencing results of the target fragment of the NR4A2-CG in the reverse direction.
